# The Potential of Modulating the Reducing Sugar Released (and the Potential Glycemic Response) of Muffins Using a Combination of a Stevia Sweetener and Cocoa Powder †

**DOI:** 10.3390/foods8120644

**Published:** 2019-12-05

**Authors:** Jingrong Gao, Xinbo Guo, Margaret A. Brennan, Susan L. Mason, Xin-An Zeng, Charles S. Brennan

**Affiliations:** 1 School of Food Science and Engineering, South China University of Technology, Guangzhou 510640, China; guoxinbo@scut.edu.cn (X.G.); xazeng@scut.edu.cn (X.-A.Z.); 2 Department of Wine, Food and Molecular Biosciences, Lincoln University, Christchurch 7647, New Zealand; margaret.brennan@lincoln.ac.nz (M.A.B.); Sue.mason@lincoln.ac.nz (S.L.M.); 3 Riddet Research Institute, Palmerston North 4442, New Zealand; 4 Overseas Expertise Introduction Center for Discipline Innovation of Food Nutrition and Human Health (111 Center), Guangzhou 510640, China

**Keywords:** muffin, in vitro starch digestibility, glycemic index, stevia, sugar replacement

## Abstract

Muffins are popular bakery products. However, they generally contain high amounts of sugar. The over-consumption of muffins may therefore result in a high calorie intake and could lead to increased health risks. For this reason, muffins were prepared substituting sucrose with two levels of a base of stevia (Stevianna^®^). In addition, cocoa powder and vanilla were added to the muffin formulation with and without Stevianna^®^ to mask any potential off flavors. Results illustrate that muffins with 50% Stevianna^®^ replacement of sucrose were similar to the control samples in terms of volume, density and texture. However, replacement of sugar with 100% Stevianna^®^ resulted in reductions in height (from 41 to 28 mm), volume (from 63 to 51 mL), and increased firmness (by four-fold) compared to the control sample. Sugar replacement significantly reduced the in vitro predictive glycemic response of muffins (by up to 55% of the control sample). This work illustrates the importance of sugar in maintaining muffin structure as well as controlling the rate of glucose release during simulated digestions.

## 1. Introduction

In recent years, consumers have gained an increasing awareness regarding the effect of dietary carbohydrates on the nutritional quality of foods. In particular, attention has been focused on the relationship between the various types of carbohydrate containing foods and the different postprandial glucose responses by these foods post ingestion [1,2,3,4,5,6,7]. The glycemic index (GI) is a physiological classification widely accepted for carbohydrate foods based on their ability to raise the concentration of glucose in the blood [7,8,9]. Bakery foods, muffins for example, are regarded as a high glycemic impact food due to the high concentration of sugar contained in the muffins. Previous research [10,11] has shown that the over-consumption of sucrose can lead to a number of metabolic complications including hyperinsulinemia, hyperglycemia, hypertension and insulin resistance, as well as being related to dyslipidemia and ectopic lipid deposition in healthy subjects with diabetes [12]. Indeed, high GI food products are quickly digested and their carbohydrate is rapidly absorbed, resulting in higher blood glucose levels [13]. On the contrary, the health benefits of the low GI products are thought to be derived from the slower the rate of carbohydrate absorption, consequently leading to a gradual rise in blood glucose level and better glycemic control [14].

The food industry has focused on reducing the calorific content of food to promote a healthier diet. Therefore, different natural sweeteners have been used in sugar-reduced or sugar-free products based on their multiple potential health benefits and functional properties, including maintaining sweetness and acceptable texture [15,16,17,18].

Steviol glycosides have been extracted and purified from the leaves of *Stevia rebaudiana* Bertoni, commonly known as stevia; they are naturally sweet-tasting, have good solubility in water, good temperature and pH stability [19,20,21] as well as having no calorific value [22], allowing them to be used as a sugar substitute or natural sweetener. Stevioside and rebaudioside A are the major glycoside constituents responsible for sweetness and are the most abundant glycosides in the *Stevia rebaudiana* Bertoni plant [23,24,25]. They are very useful as a food additive due to their relative sweetness being 250–300 times sweeter than table sugar [26].

Extracts from stevia have broad health-promoting properties for blood glucose and insulin levels in human studies [27]. Steviol glycosides are not hydrolyzed by human digestive enzymes of the mouth, stomach, and small intestine [28]. However, rebaudioside A and stevioside are hydrolyzed (in vitro and in vivo) to aglycone steviol by colon microflora through the successive removal of glucose units [29]. Chang et al. [27] reported that insulin sensitivity is increased due to stevia consumption in rodent models, and thus does not increase blood glucose and insulin levels [22]. Furthermore, previous work has found that a reduction in the predicted glycemic response was observed due to 50% or 100% replacement of sucrose with Stevianna^®^ in muffins during in vitro digestion experiments [30]. Therefore, stevia has the potential to be a low-cost natural sweetener due to important pro-health properties, such as being non-calorific, non-fermentable and non-toxic as well as having a high-intensity sweetness [31], and it is also recommended as a treatment for diabetics and obese persons [23].

However, several studies have shown that the utilization of stevia as a sugar replacer in baking leads to a negative effect on appearance, compactness, moisture and texture of the bakery products structure [17,32,33]. These results have indicated that stevia is not acceptable to replace sucrose completely in bakery products as stevia exhibits high-intensity sweetness but does not possess the necessary bulking characteristics [34]. That is why Stevianna^®^ (product code ST001 SE supplied by Stevianna^®^ NZ) is used for our study, as it incorporates rebaudioside A (98% steviol glycoside; 1%) with erythritol (99%).

Erythritol is a four-carbon sugar alcohol or polyol with approximately 60% to 80% of the sweetness of sucrose [35]. It is not only a sweetener but also a bulking agent, and thus can be used as a sugar replacer in bakery products. Partial replacement of sucrose with erythritol had no negative influence on physical quality characteristics in a baked product [34,36]. In addition, previous studies reported that erythritol is useful as a non-glycemic and low-calorie sweetener that is safe for diabetics [37,38]. Erythritol has been demonstrated to have a small molecular size, thus it is rapidly absorbed by the small intestine and does not undergo systemic metabolism by the human body [37,39]. Some research has shown that the combination of a high-intensity sweetener with bulking agents or fibers in sugar-reduced formulations of food resulted in bakery products with acceptable physical quality [26,29,40,41].

None of these previous studies assessed a complex food sweetener to replace traditional sugar in bakery products. The aim of the study was to evaluate the replacement of sugar with Stevianna^®^ (1 × sweetness of sucrose) and the addition of cocoa powder and/or vanilla to muffins for their physical properties and glycemic response, compared with a control muffin formulation with no added Stevianna^®^, cocoa powder, or vanilla.

## 2. Materials and Methods

### 2.1. Raw Materials

Wheat flour (Medal Premium baker flour, Champion, Auckland, New Zealand), white sugar (Chelsea, Auckland, New Zealand), baking powder (Edmonds, Christchurch, New Zealand), iodized table salt (Cerebos, Auckland, New Zealand), skim milk powder (Pams, Auckland, New Zealand), 100% cocoa powder (Cadbury, Dunedin, New Zealand), vanilla (Hansells, Sydney, Australia), canola oil (Pams, Auckland, New Zealand), and fresh eggs were purchased from a local supermarket and tap water was used. Muffins were prepared containing 0%, 50% and 100% Stevianna^®^ (produce code ST001_SE; Stevianna^®^, Auckland, New Zealand) as a replacement for sucrose. Stevianna^®^ utilizes Reb-A 98% steviol glycoside as the main sugar substitute along with erythritol.

### 2.2. Muffin Preparation

The muffin recipe was adapted from a previous study [30] and is given in Table 1. The Stevianna^®^ was dissolved in the water and mixed with liquid whole egg and oil. After that, the dry ingredients were added into the liquid components and mixed for 5 min. The batter was poured into a paper baking case in a muffin pan. The muffins were baked for 18 min in a preheated Simpson Gemini Atlas series oven at 180 °C set to fan bake. Baked muffins were cooled at room temperature for 1 h, then packed in plastic resealable bags and stored in a refrigerator at 4 °C until physical analysis.

### 2.3. Muffin Height

The muffin product was taken out from the paper baking case, and the muffin height was measured with an electronic caliper (INSIZE) from the highest point of the muffin to the bottom of the muffin.

### 2.4. Moisture Content

A domestic kitchen food chopper (Zyliss^®^) was used to crush and homogenize the muffin (crust and crumb) of each formulation. Approximately 4 g was dried in an air oven at 105 °C for 16 h, until no further weight change.

The moisture content (MC) was calculated using the following equation:

MC (%) = (W_before drying_ − W_after drying_/W_before drying_) × 100 (1)

where W denotes weight (g).

### 2.5. Muffin Volume

The volume of the muffins was measured by the rapeseed displacement method. Each muffin was placed in a plastic beaker of known volume (total volume, Vt), and the remaining space in the plastic beaker was then filled with rapeseed; the volume of the rapeseed required (Vs) was then determined by graduated cylinder. Muffin volume was calculated as the difference between the total volume and volume of rapeseed—the muffin volume = Vt − Vs [36].

### 2.6. Muffin Texture

A texture analyzer (TA.XT. Plus, Stable Microsystems, Surrey, UK) was used to measure the texture profile of muffins in terms of the firmness and springiness of the samples. The samples were compressed to a strain of 25% of the original height using a 75 mm cylindrical probe and a 50 kg load cell, and a test speed of 1.0 mm/s was used. Data was obtained from the Texture expert software (Stable Microsystems, Surrey, UK). Firmness and springiness values were calculated as the overall force of compression required and the resistance post compression.

### 2.7. Muffin Total Starch

Total starch analysis was carried out according to the official American Association of Cereal Chemists method 76.13 [42], using Megazyme (Bray, Dublin, Ireland) total starch kit.

### 2.8. In Vitro Predictive Glycemic Response Digestion Analysis

The procedure used for the determination of potential glycemic response is the same as that reported previously by [30]. This procedure measures the breakdown of carbohydrates to sugars by the action of amylase enzymes added to the baked muffin. Whole muffins were chopped with a domestic kitchen food chopper (Zyliss^®^) to stimulate particle size reduction which occurs during natural mastication for at least one minute of steady chopping until a fine crumb was achieved. A 3.5 g sample was used to determine the predictive glycemic response.

Triplicate samples of product (approximate 1 g of cooked muffin) were each placed into the 60 mL plastic pots and 30 mL of distilled water added, and duplicate blank samples. These pots were inserted to a pre-heated 15 place magnetic heated stirring block (IKAMAG^®^ RT15, IKA^®^-WERKE Gmblt & Co., Staufen, Germany) preheated to 37 °C, on each pot one magnetic stirrer, followed by 0.8 mL of 1 M aqueous HCl. Then, 1 mL of a 10% pepsin (Acros Organics, New Jersey, NJ, USA CAS: 901-75-6) solution in 0.05 M HCl was added in order to replicate gastric digestion. The sample was incubated at 37 °C for 30 min with slow constant stirring (130 rpm) to simulate gastric digestion conditions. In vitro stomach digestion was halted by the addition of 2 mL NaHCO_3_. Small intestine digestion was mimicked by the addition of 5 mL 0.1 M Na maleate buffer pH 6. An aliquot (1 mL) was withdrawn (Time 0) and added to 4 mL absolute ethanol to stop any further enzyme reaction. A 0.1 mL dose of amyloglucosidase (A.niger, Megazyme, E-AMGDF; 3260 U/mL) was added to prevent end-product inhibition of pancreatic amylase. A 5 mL 2.5% pancreatin (EC: 232-468-9, CAS: 8049-47-6, activity: 42362 FIP-U/g, Applichem GmbH, Darmstadt, Germany) in 0.1 M Na maleate buffer pH 6 followed by the volume being made to 53 mL with continued stirring and heat maintained at 37 °C for 120 min. Triplicate 1 mL aliquots were withdrawn at 0, 20, 60, 120 min and added to 4 mL absolute ethanol. Reducing sugar content was analyzed by dinitrosalicyclic (DNS) colorimetry, and the area under the curve (AUC) was calculated by dividing the graph into trapezoids as described elsewhere [30]. The reducing sugar content was regarded as an indicator for the predictive glycemic response.

### 2.9. Statistical Analyses

All analyses were conducted in triplicate. Analysis of variance (one-way ANOVA) was performed on the data, and Tukey’s comparison test (*p* < 0.05) was used to determine the significance. These analyses were performed using Minitab (Minitab Pty Ltd., Sydney, Australia).

## 3. Results and Discussion

### 3.1. Moisture Content

**Figure 1:**
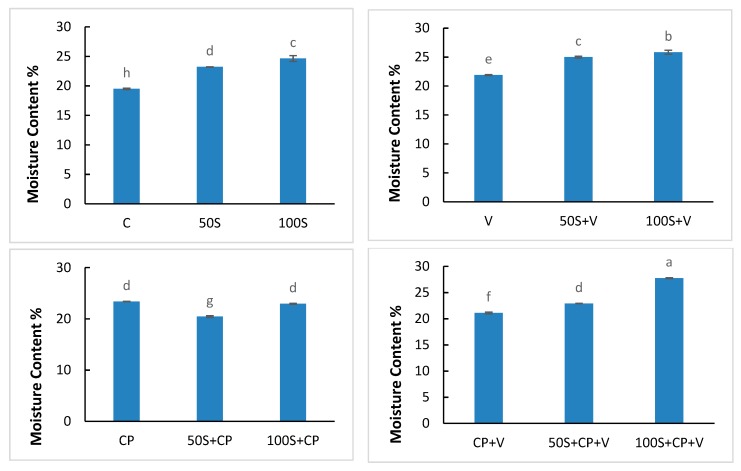
Moisture content for muffins of formulation made from two levels of Stevianna without/with cocoa powder and/or vanilla. Control (C); Vanilla (V); Cocoa Powder (CP); Cocoa + Vanilla (CP + V); 50% Stevianna (50S); 50% Stevianna + Vanilla (50S + V); 50% Stevianna + Cocoa (50S + CP); 50% Stevianna + Cocoa + Vanilla (50S + CP + V); 100% Stevianna (100S); 100% Stevianna + Vanilla (100S + V); 100% Stevianna + Cocoa (100S + CP); 100% Stevianna + Cococa + Vanilla (100S + CP + V). Values with different letters are significantly different to one another *p* < 0.05.

Table 2 shows that the moisture content of muffin samples ranged from 19% to 27%. The moisture content of the muffin samples produced was higher when cocoa powder or/and vanilla was used. In addition, Figure 1 shows that moisture content values increased significantly (*p* < 0.05) when sucrose was replaced by Stevianna^®^—in particular the moisture content of 100% Stevianna^®^ samples were higher than the full-sucrose muffin samples. Sucrose plays an important role in water retention that results in reduced moisture loss during the baking of the muffins [43]. However, the moisture content increased when sucrose was replaced because the Stevianna^®^ acted as a humectant and prevented water from escaping during baking. Research using other types of sugar replacers has shown similar results. Martínez-Cervera et al. [44] used erythritol in muffins for its water retention properties. Ghosh and Sudha [45] showed that the use of the polyol sorbitol was reflected in a significantly higher moisture content (*p* < 0.05). Due to the high water-binding capacity of formulations with carbohydrate-based sugar replacers, a greater amount of water is required in cereal products.

Moisture content in bakery products is an important factor as it has a direct impact on the texture attributes and a strong correlation has been found between moisture content and firmness [46]. As can be seen from the Table 2, muffin firmness increased as moisture content increased. As reported by Rößle et al. [47], this must be related to the replacement of the sugar by Stevianna^®^, affecting the formation of muffin structure.

### 3.2. The Impact of Sugar Replacement on Product Physico-Chemical Characteristics

**Figure 2:**
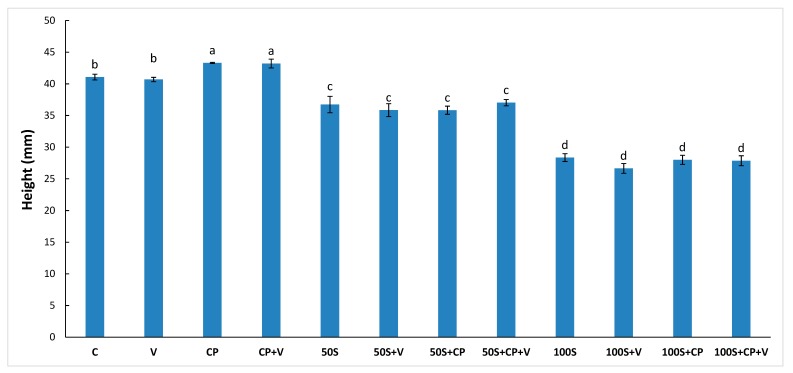
Effect of Stevianna without/with cocoa powder and/or vanilla on the height of muffin. Control (C); Vanilla (V); Cocoa Powder (CP); Cocoa + Vanilla (CP + V); 50% Stevianna (50S); 50% Stevianna + Vanilla (50S + V); 50% Stevianna + Cocoa (50S + CP); 50% Stevianna + Cocoa + Vanilla (50S + CP + V); 100% Stevianna (100S); 100% Stevianna + Vanilla (100S + V); 100% Stevianna + Cocoa (100S + CP); 100% Stevianna + Cococa + Vanilla (100S + CP + V). Values with different letters are significantly different to one another *p* < 0.05.

**Figure 3:**
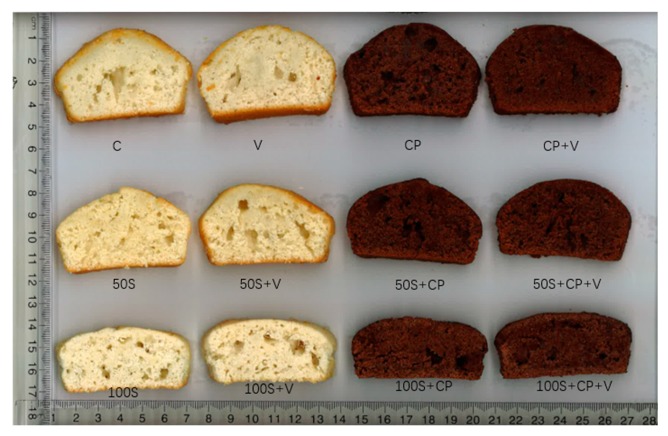
Effect of two levels of Stevianna with/without cocoa powder and/or vanilla in muffins: Control (C); Vanilla (V); Cocoa Powder (CP); Cocoa + Vanilla (CP + V); 50% Stevianna (50S); 50% Stevianna + Vanilla (50S + V); 50% Stevianna + Cocoa (50S + CP); 50% Stevianna + Cocoa + Vanilla (50S + CP + V); 100% Stevianna (100S); 100% Stevianna + Vanilla (100S + V); 100% Stevianna + Cocoa (100S + CP); 100% Stevianna + Cococa + Vanilla (100S + CP + V).

The height of the muffins prepared with the different levels of Stevianna^®^ with/without cocoa powder and/or vanilla is shown in Figure 2. The full-sucrose muffin was significantly higher (*p* < 0.05) than the muffins that were prepared using Stevianna^®^. The lowest height was found in the 100% Stevianna^®^ muffin samples. The full-sucrose muffin with cocoa powder and/or vanilla group had a greater height than the control and other samples (Figure 2). These results indicate that the decrease in muffin height was associated with an absence of interconnectivity of a more compact structure and with a low number of air cells for levels of sucrose replacement higher than 50% (Figure 3).

Photographs of vertical cross-sections of the different muffin formulations are shown in Figure 3. As the Stevianna^®^ content increased, in the formulations, the air bubbles became smaller and the air channels gradually diminished. This could be due to the fact that muffins with a full sucrose content gained an increased number of air bubbles during the beating of the batter, and these air bubbles are then expanded by carbon dioxide and water vapor pressure generated during baking, resulting in the formation of air channels, which influence the texture of the finished muffin product. The lack of air channels as the sucrose was replaced may also be associated with earlier thermosetting of the batter during the heating process in the oven, therefore, not allowing enough time for bubble expansion and formation of air channels [43,44]. Martínez-Cervera et al. [44] also found that the number of small air bubbles increased, air channels diminished, and circular bubbles increased with an increase in sucrose replacement by polydextrose and sucralose in a muffin product.

**Figure 4:**
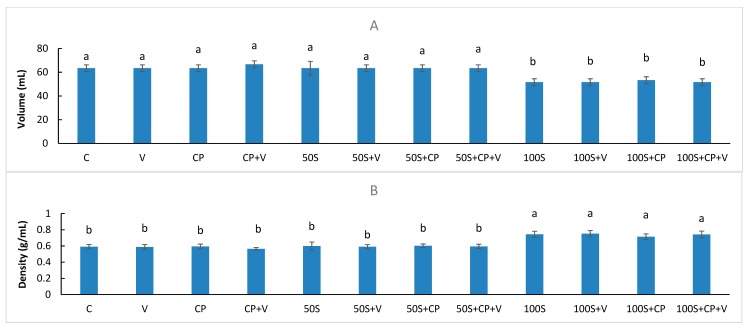
Volume (**A**) and density (**B**) values for muffins containing two levels of Stevianna as sugar replacer with or without cocoa powder and vanilla. Control (C); Vanilla (V); Cocoa Powder (CP); Cocoa + Vanilla (CP + V); 50% Stevianna (50S); 50% Stevianna + Vanilla (50S + V); 50% Stevianna + Cocoa (50S + CP); 50% Stevianna + Cocoa + Vanilla (50S + CP + V); 100% Stevianna (100S); 100% Stevianna + Vanilla (100S + V); 100% Stevianna + Cocoa (100S + CP); 100% Stevianna + Cococa + Vanilla (100S + CP + V). Values with different letters are significantly different to one another *p* < 0.05.

The volume of the muffin is an important indicator of air bubble expansion during baking and consequently also of the porous structure of the product. The volumes of muffins prepared with different levels of Stevianna^®^ with/without and/or vanilla along with the control muffin are presented in Figure 4A. The samples with 100% Stevianna^®^ muffin group had significantly lower volumes (*p* < 0.05) compared to those of the full-sucrose muffin products. Muffin density appeared to be negatively correlated with muffin volume (Figure 4B). The density of the muffins was calculated from mass and volume after baking. Table 2 illustrates that when sugar was completely substituted with Stevianna^®^, there was a significant increase (*p* < 0.05) in muffin density. Additionally, product quality characteristics such as springiness and firmness were greatly affected (Table 2). These results indicate that an increase in the level of Stevianna^®^ had an adverse effect on volume, density and texture of the muffin. Manisha et al. [26] also reported that replacement of sucrose with 100% stevioside and liquid sorbitol caused a significant deterioration in quality which decreased volume and resulted in a firmer texture in cake properties.

A function of sugar during cake baking is that it delays starch gelatinization, thus contributing to the aeration of the batter and the optimum quality of sugar will affect formation of the cake structure and improve crumb texture and tenderness [26]. The decrease in sugar-free muffin expansion is the result of less air bubble incorporation and reduced air holding capacity during baking [48]. In addition, starch gelatinization temperature seems to contribute to volume development due to different interactions between the Stevianna^®^ and starch and proteins of the batter, and these interactions affect starch gelatinization and protein denaturation temperatures. These results are in agreement with Ronda et al. [49]’s findings which showed that a decrease in starch gelatinization and protein denaturation temperatures in sorbitol cakes is expected to cause a premature thermosetting of protein or starch matrix—this process will start at the crust due to direct contact with the heating medium. Therefore, this lowers the heat transfer rate, and produces a vapor pressure build-up, resulting in inadequate expansion of individual bubbles. Additionally, Ronda et al. [49] found that high-fructose corn syrup (HFCS) mainly contributed to the early gelatinization of starch during the baking process and restricted the volume of baked products compared to sucrose.

However, the 50% Stevianna^®^ used had no significant effect on the volume and density of muffin compared to the full-sucrose muffin samples (Figure 4). These results suggest that muffin samples containing half the amount of Stevianna^®^ have a similar ability, compared with muffins with full sucrose, to retain air. These results are consistent with those of Lin et al. [38], who found no significant differences among the volume estimates for 50% erythritol cakes. Furthermore, the addition of the 50% Stevianna^®^ in muffin samples exhibited a texture close to that of the full-sucrose muffin samples (Table 2), which conferred an appearance of firmness and springiness. The results were consistent with previous research [30].

### 3.3. The Impact of Sugar Replacement on the In Vitro Predictive Glycemic Response

The total starch of modified muffins was measured and compared with the control sample (Table 2). Compared to the control muffin, 50% or 100% sucrose replacement with Stevianna^®^ with added cocoa powder samples had significantly lower amounts of total starch. Similar levels of total starch were observed in control and full-sucrose muffin samples—50% and 100% Stevianna^®^ with/without cocoa powder and/or vanilla muffin samples. Thus, the presence of cocoa powder with Stevianna^®^ in muffin had a significant effect on total starch contents.

**Figure 5:**
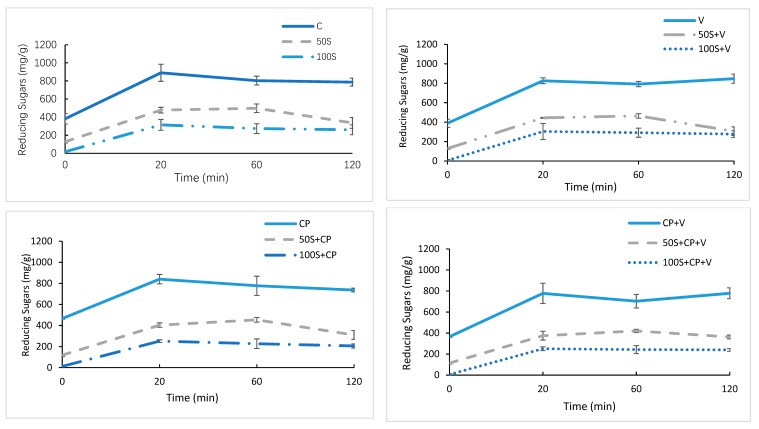
Amount of reducing sugars released per g of food material during in vitro digestion. Control (C); Vanilla (V); Cocoa Powder (CP); Cocoa + Vanilla (CP + V); 50% Stevianna (50S); 50% Stevianna + Vnilla (50S + V); 50% Stevianna + Cocoa (50S + CP); 50% Stevianna + Cocoa + Vanilla (50S + CP + V); 100% Stevianna (100S); 100% Stevianna + Vanilla (100S + V); 100% Stevianna + Cocoa (100S + CP); 100% Stevianna + Cococa + Vanilla (100S + CP + V).

The effects of Stevianna^®^ on in vitro starch digestion in muffin and chocolate muffin products were investigated by measuring the glucose released during starch digestion. Figure 5 shows the reducing sugars curves of two levels of Stevianna^®^ with/without cocoa powder and/or vanilla muffin samples that were compared with full-sucrose with/without cocoa powder and/or vanilla samples, respectively. These two levels of Stevianna^®^ used in this study were found to decrease reducing sugars released by digestive enzymes, compared with the full-sucrose muffin samples. The rate and extent of reducing sugars released were the highest in the control muffin, followed by 50% Stevianna^®^ with/without cocoa powder and/or vanilla muffin products, and 100% Stevianna^®^ with/without cocoa powder and/or vanilla muffins (Figure 5). In particular, muffins with Stevianna^®^ showed a significant decrease in terms of reducing sugars released throughout the 120 min starch digestion process.

**Figure 6:**
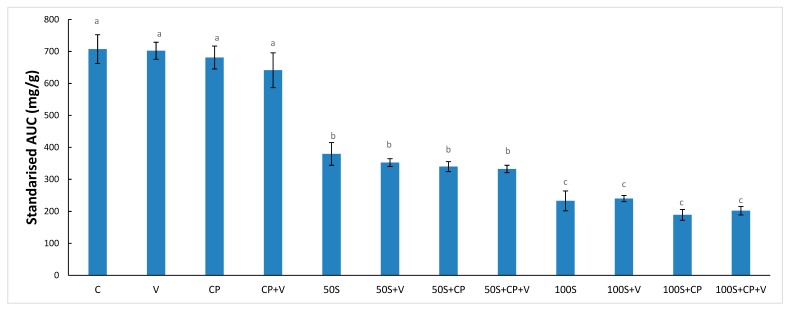
Values for area under the curve (AUC) comparing the control and other low-sugar muffins made with two levels of Stevianna with/without cocoa powder and/or vanilla. Control (C); Vanilla (V); Cocoa Powder (CP); Cocoa + Vanilla (CP + V); 50% Stevianna (50S); 50% Stevianna + Vanilla (50S + V); 50% Stevianna + Cocoa (50S + CP); 50% Stevianna + Cocoa + Vanilla (50S + CP + V); 100% Stevianna (100S); 100% Stevianna + Vanilla (100S + V); 100% Stevianna + Cocoa (100S + CP); 100% Stevianna + Cococa + Vanilla (100S + CP + V). Values with different letters are significantly different to one another *p* < 0.05.

The total area under the hydrolysis curve (AUC) relates the total glucose release to the digestion time of 120 min. The concentration of the Stevianna^®^ had a significant effect on the AUC values (*p* < 0.05), which demonstrated that the replacement of sucrose with 100% Stevianna^®^ resulted in the lowest AUC value of muffin samples in a dose response (Figure 6). It is of interest that the additions of vanilla and/or cocoa powder with muffin production did not lead to a significant reduction of in vitro digestion values compared to the full-sucrose—50% Stevianna^®^, and 100% Stevianna^®^ samples, respectively. These results are consistent with the previous report by Gao et al. [30].

This study did not focus on the impact of sweeteners on in vitro starch digestion analysis of bakery products. However, several research projects have been designed to test the effects of the stevia or erythritol on postprandial glucose and insulin levels in vivo and in vitro digestion methods as compared to sucrose [50,51].

The breakdown or disruption of starch granules that results from salivary amylase causes a greater susceptibility of the granule to further enzyme degradation. This process will lead to more readily digestible starch, and hence create a higher blood glucose response [52]. The level of postprandial blood glucose is a major factor in predicting the profile of insulin resistance. Alizadeh et al. [50] found that there were differing effects on postprandial blood insulin levels that were dependent on the type and amount of sweetener consumed. The effect of the consumption of beverages containing stevia has been tested by measuring the in vivo glycemic impact [53], and it was found that postprandial glucose and insulin levels were significantly reduced in the stevia beverages compared to the sucrose beverages. These effects on postprandial glucose levels are mainly due to the lack of calories and carbohydrate content of Stevianna^®^, and thus there are no reducing sugars released. A similar trend has been observed in that the postprandial insulin levels were reduced in stevia ice cream samples compared to full-sucrose ice cream samples [50], and this is most likely due to the functional properties of stevia that results in no contribution to the available carbohydrate and glycemic response in food products. In addition, Roberts and Renwick [54] illustrated that steviol glycosides are not readily absorbed by the upper small intestine when it is administered orally to normal rat or human subjects. There are no human digestive enzymes present in the small intestine to hydrolyze the β-glycosidic linkages, resulting in limited small intestine digestion.

Lin et al. [36] illustrated that 0%–100% sugar replacement with erythritol in cookies decreased the carbohydrate contents by in vivo digestion. Since the calorie value of erythritol is approximately 0.4 kcal/g [39], it provides no energy to the body and thus it is not systemically metabolized nor fermented in the colon [37]. It has been suggested that the consumption of erythritol does not raise postprandial glycemic and insulin levels by oral ingestion in healthy human subjects [28]. In a previous study [39], more than 90% of erythritol is rapidly absorbed by the small intestine when eaten and is excreted unchanged in the urine.

The Stevianna^®^ used in our study was composed of rebaudioside A (stevia) and erythritol and, therefore, the observations made are consistent with those made by the above studies. Our experiment results showed that under in vitro conditions a lower reducing sugar liberation took place when sucrose was replaced by Stevianna^®^ in muffins, and consequently this can be beneficial to as it will decrease the postprandial blood glucose. Additionally, it is probable that the intake of these muffins decreases the rate of intestine absorption of glucose and delays gastric emptying.

## 4. Conclusions

The stevia-containing product, Stevianna^®^, has been shown to be a suitable sucrose replacement for a low-sucrose formulation of muffins. The results showed that 50% sugar replacement with Stevianna^®^ had similar physical quality characteristics in terms of volume, density and texture to a control muffin. However, when the sugar was replaced by 100% Stevianna^®^, the muffin quality showed a reduction in volume, an increase in textural firmness and a correspondingly high density of the product when compared to the control muffin samples. Furthermore, Stevianna^®^ was able to simulate sucrose functionality in muffins, producing an increase in moisture content in comparison with the full-sucrose muffins. The negative effect of Stevianna^®^ on muffin properties can be associated with the fact that as the Stevianna^®^ level was raised, it led to a reduction of air bubble expansion during the heating process (possibly due to the weakening of the starch–protein–sugar interface of the muffin, allowing for greater structural collapse) and thus a corresponding reduction in height. This research illustrates that Stevianna^®^ is a major factor impacting on the physical characteristics of muffins. The addition of cocoa powder and/or vanilla did not affect the quality of muffins significantly.

In relation to the nutritional quality of the muffin products, the effect of Stevianna^®^ inclusion on the predicted glycemic impact as determined by in vitro digestion illustrated the role of sugar in elevating the glycemic response during digestion. The replacement of sugar with increasing levels of Stevianna^®^ was found to significantly decrease the potential glycemic response values, and this is most likely to be attributed to the fact that Stevianna^®^ was not degraded into glucose units and acted as an inert filler within the muffin samples. Therefore the inclusion of cocoa powder and/or vanilla powder did not have a significant change to the predicted glycemic response values of the muffins.

The breakdown or disruption of starch granules that results from salivary amylase causes a greater susceptibility of the granule to further enzyme degradation. This process will lead to more readily digestible starch, and hence create a higher blood glucose response [52]. The level of postprandial blood glucose is a major factor in predicting the profile of insulin resistance. Alizadeh et al. [50] found that there were differing effects on postprandial blood insulin levels that were dependent on the type and amount of sweetener consumed. The effect of the consumption of beverages containing stevia has been tested by measuring the in vivo glycemic impact [53], and it was found that postprandial glucose and insulin levels were significantly reduced in the stevia beverages compared to the sucrose beverages. These effects on postprandial glucose levels are mainly due to the lack of calories and carbohydrate content of Stevianna^®^, thus there are no reducing sugars released. A similar trend has been observed in that the postprandial insulin levels were reduced in stevia ice cream samples compared to full-sucrose ice cream samples [50], and this is most likely due to the functional properties of stevia that results in no contribution to the available carbohydrate and glycemic response in food products. In addition, Roberts and Renwick [54] illustrated that steviol glycosides are not readily absorbed by the upper small intestine when it is administered orally to normal rat or human subjects. There are no human digestive enzymes present in the small intestine to hydrolyze the β-glycosidic linkages, resulting in limited small intestine digestion.

Lin et al. [36] illustrated that 0%–100% sugar replacement with erythritol in cookies decreased the carbohydrate contents by in vivo digestion. Since the calorie value of erythritol is approximately 0.4 kcal/g [39], it provides no energy to the body and thus it is not systemically metabolized nor fermented in the colon [37]. It has been suggested that the consumption of erythritol does not raise postprandial glycemic and insulin levels by oral ingestion in healthy human subjects [28]. In a previous study [39], more than 90% of erythritol is rapidly absorbed by the small intestine when eaten and is excreted unchanged in the urine.

Finally, it can be seen that a partial replacement of Stevianna^®^ for sucrose with/without cocoa powder and/or vanilla in muffins gave a product with quality characteristics close to that of the full-sucrose muffin sample. At the same time, the reduction in potential glycemic response values was greater than would have been expected with 50% sucrose reduction and consequently providing a quality muffin that produces a lowered postprandial response with the potential associated health benefits.

## Figures and Tables

**Table 1:** Formulas for muffins at two Stevianna levels, with or without cocoa powder and/or vanilla.

Formulation ^a^	C	V	CP	CP + V	50S	50S + V	50S + CP	50S + CP + V	100S	100S + V	100S + CP	100S + CP + V
Ingredients	Mass (g)											
Wheat flour	138.4	138.4	115.3	115.3	138.4	138.4	115.3	115.3	138.4	138.4	115.3	115.3
Sugar	92.2	92.2	92.2	92.2	46.1	46.1	46.1	46.1	0	0	0	0
Baking powder	6.5	6.5	6.5	6.5	6.5	6.5	6.5	6.5	6.5	6.5	6.5	6.5
Salt	1.4	1.4	1.4	1.4	1.4	1.4	1.4	1.4	1.4	1.4	1.4	1.4
Skim milk powder	8.7	8.7	8.7	8.7	8.7	8.7	8.7	8.7	8.7	8.7	8.7	8.7
Oil	77.6	77.6	77.6	77.6	77.6	77.6	77.6	77.6	77.6	77.6	77.6	77.6
Liquid whole egg	34.6	34.6	34.6	34.6	34.6	34.6	34.6	34.6	34.6	34.6	34.6	34.6
Top water	97.6	97.6	97.6	97.6	97.6	97.6	97.6	97.6	97.6	97.6	97.6	97.6
Cocoa powder	0	0	23.1	23.1	0	0	23.1	23.1	0	0	23.1	23.1
Vanilla	0	3	0	3	0	3	0	3	0	3	0	3
Stevia	0	0	0	0	46.1	46.1	46.1	46.1	92.2	92.2	92.2	92.2

^a^ Sample name of formulation: Control (C); Vanilla (V); Cocoa Powder (CP); Cocoa+Vanilla (CP + V); 50% Stevianna (50S); 50% Stevianna + Vanilla (50S + V); 50% Stevianna + Cocoa (50S + CP); 50% Stevianna + Cocoa + Vanilla (50S + CP + V); 100% Stevianna (100S); 100% Stevianna + Vanilla (100S + V); 100% Stevianna + Cocoa (100S + CP); 100% Stevianna + Cococa + Vanilla (100S + CP + V).

**Table 2:** Effect of Stevianna on texture profile analysis and total starch in muffins with or without cocoa powder and/or vanilla.

Product	Firmness (g)	Springiness (%)	Total Starch (%)
C	746.06 ± 44.10 ^b^	51.29 ± 0.44 ^ab^	26.83 ± 1.92 ^abc^
V	763.51 ± 51.48 ^b^	51.66 ± 0.09 ^a^	27.93 ± 0.42 ^ab^
CP	680.99 ± 30.33 ^b^	49.26 ± 0.54 ^ab^	26.14 ± 0.60 ^abcd^
CP + V	662.97 ± 68.46 ^b^	49.99 ± 0.43 ^ab^	24.43 ± 1.06 ^bcde^
50S	906.07 ± 111.09 ^b^	51.51 ± 0.62 ^ab^	28.50 ±0.85 ^a^
50S + V	1102.18 ± 102.10 ^b^	51.49 ± 0.78 ^a^	29.03 ± 0.36 ^a^
50S + CP	987.03 ± 68.00 ^b^	48.67 ± 0.52 ^a^	22.72 ± 0.39 ^de^
50S + CP + V	890.78 ± 76.18 ^b^	49.59 ± 0.54 ^b^	23.40 ± 0.09 ^cde^
100S	4512.78 ± 399.65 ^a^	45.07 ± 0.71 ^c^	26.60 ± 0.94 ^abc^
100S + V	4419.70 ± 409.69 ^a^	45.44 ± 0.56 ^c^	29.09 ± 2.56 ^a^
100S + CP	3868.00 ± 300.87 ^a^	44.74 ± 1.12 ^c^	22.62 ± 1.42 ^e^
100S + CP + V	3839.94 ± 522.34 ^a^	43.11 ± 1.36 ^c^	26.17 ± 1.14 ^abcd^

Control (C); Vanilla (V); Cocoa Powder (CP); Cocoa+Vanilla (CP + V); 50% Stevianna (50S); 50% Stevianna + Vanilla (50S + V); 50% Stevianna + Cocoa (50S + CP); 50% Stevianna + Cocoa + Vanilla (50S + CP + V); 100% Stevianna (100S); 100% Stevianna + Vanilla (100S + V); 100% Stevianna + Cocoa (100S + CP); 100% Stevianna + Cococa + Vanilla (100S + CP + V). All measurements are the mean values ± SD of triplicate determinations. Means in the same column with different letters are significantly different (*p* < 0.05).
